# The Prevention of Venereal Diseases

**Published:** 1919

**Authors:** 


**The Prevention of Venereal Diseases.** By OTTO MAY, M.A., M.D., M.R.C.P. Pp. 240. London: Oxford Medical Publications. 1918. Price 7s. 6d. net.

During this time of national appreciation of the urgency of the prevention and treatment of venereal diseases Otto May's book appears opportunely.

It presents the case fairly and in judicious language, and should be of service to anyone who is working in an attempt to combat venereal diseases, as well as to those who wish to understand what is being done, or what may be accomplished for that end.

The chapter on " Personal Prevention " is of special value.

